# *SUSD2* expression in high-grade serous ovarian cancer correlates with increased patient survival and defective mesothelial clearance

**DOI:** 10.1038/oncsis.2016.64

**Published:** 2016-10-24

**Authors:** J N Sheets, M Iwanicki, J F Liu, B E Howitt, M S Hirsch, J A A Gubbels, R Drapkin, K A Egland

**Affiliations:** 1 Cancer Biology Research Center, Sanford Research, Sanford School of Medicine of the University of South Dakota, Sioux Falls, SD, USA; 2 Department of Cell Biology, Dana-Farber Cancer Institute, Boston, MA, USA; 3 Department of Medical Oncology, Dana-Farber Cancer Institute, Harvard Medical School, Boston, MA, USA; 4 Department of Pathology, Brigham and Women's Hospital, Harvard Medical School, Boston, MA, USA; 5 Department of Pathology, Brigham and Women's Hospital, Harvard Medical School, Boston, MA, USA; 6 Department of Biology, Augustana College, Sioux Falls, SD, USA; 7 Department of Obstetrics and Gynecology, Ovarian Cancer Research Center, University of Pennsylvania Perelman School of Medicine, Philadelphia, PA, USA

## Abstract

The cause of death among the majority of epithelial ovarian cancer (EOC) patients involves passive dissemination of cancer cells within the peritoneal cavity and subsequent implantation of cancer spheroids into adjacent organs. Thus, it is important to identify the factors that mediate EOC metastasis and implantation, including clearance of the mesothelium. *Sushi domain containing 2 (SUSD2)* encodes a type I transmembrane protein containing several functional domains inherent to adhesion molecules. Immunohistochemical analysis determined the presence of SUSD2 in several subtypes of EOC, with the strongest staining observed in high-grade serous ovarian carcinomas (HGSOCs). A high-density, clinically annotated HGSOC tissue microarray was stained with an anti-SUSD2 antibody. Patients with tumors that had a low percentage of SUSD2 staining cells had a shorter median survival (31.7 months) compared with patients who had tumors with extensive SUSD2 staining (49.1 months; *P*-value=0.0083). To investigate the role of SUSD2 in HGSOCs, stable OVCAR3, OVSAHO and KURAMOCHI cell lines were established with knockdown (KD) or non-targeting (NT) of SUSD2. Boyden chamber and wound-healing assays demonstrated that OVCAR3, OVSAHO and KURAMOCHI SUSD2-KD cells migrated at significantly higher rates compared with their SUSD2 NT counterpart cell lines. Quantitative reverse transcription–PCR and western immunoblot analysis indicated an inverse relationship between SUSD2 and well-characterized mesenchymal proteins, including Twist-1, Zeb-1, N-cadherin, STEAP1, AHNAK, Snail-1, COL5A2 and Snail-3 in OVCAR3, OVSAHO and KURAMOCHI cell line models. In addition, OVCAR3 and KURAMOCHI SUSD2-KD spheroids displayed increased mesothelial clearance ability compared with cells that express endogenous levels of SUSD2. These data suggest that SUSD2 has a role in the inhibition of mesothelial clearance, which is required for metastasis. Altogether, our findings indicate that SUSD2 impedes migration, epithelial-to-mesenchymal transitional and mesothelial clearance of HGSOC cells, consistent with prolonged survival of patients with *SUSD2*-expressing tumors.

## Introduction

Epithelial ovarian cancer (EOC) is the leading cause of death in gynecological malignancy and ranks fifth in mortality rates among all cancers in the United States.^1, 2^ Termed the ‘silent killer', EOC is accompanied by vague and minor symptoms during onset and initial stages of the disease progression (stages I and II), which impedes patient diagnosis at an early stage. Unfortunately, ~75% of EOC patients are diagnosed when the cancer has become widely disseminated in the peritoneal cavity (stages III and IV).^3^ The American Cancer Society estimates that ~67% of women diagnosed with ovarian cancer in 2015 will die from their disease, showing the need for early detection tools and novel approaches to treatment.^4^

The majority of EOC cases are diagnosed as high-grade serous ovarian carcinomas (HGSOCs), representing the most deadly of all EOC subtypes.^5^ Unique in its dissemination process, EOC does not typically require hematogenous intravasation/extravasation as a means of metastasis. Instead, upon reaching a certain threshold defined by many variables including growth and epithelial-to-mesenchymal transitional (EMT) events, a primary ovarian tumor will passively shed single cells and/or small aggregates of cells, leading to spheroid formation in the peritoneal cavity.^3, 6, 7^

Spheroids represent the vehicle for passive EOC dissemination, carrying malignant cells to one of many abdominal surfaces, including the omentum, peritoneum, diaphragm and small bowel mesentery, where they can form secondary nodules.^8^ *In vitro* analysis has captured live, image-based interactions between ovarian cancer spheroids and mesothelial cells, a continuous monolayer of epithelial cells designed to mimic the mesothelium that lines and protects the intraperitoneal wall of the abdominal cavity, demonstrating that spheroid-induced mesothelial clearance is required for secondary nodule formation.^9^

EMT is a well-established process that occurs in many cancers including EOC.^10^ EMT events have been implicated in the progression of HGSOCs at the point of passive exfoliation of primary tumor cells into the peritoneal cavity and spheroid formation.^11, 12^ Known as the ‘cadherin switch', cells undergoing EMT will downregulate epithelial proteins, such as E-cadherin, while simultaneously upregulating mesenchymal proteins, such as N-cadherin. This altered regulation causes epithelial cells to transition into mesenchymal-like cells, decreasing cell polarity and increasing cell motility and invasion.^13^

*Sus*hi *d*omain containing *2* (SUSD2) was identified by a cDNA library enriched for genes that encode membrane and secreted proteins that are highly expressed in cancer cells with minimal expression in normal tissues.^14^ SUSD2 is a type I transmembrane protein that contains a somatomedin B, AMOP, von Willebrand factor type D and Sushi domains, which are frequently found in molecules associated with cell–cell and cell–matrix adhesion. In a recent publication, our laboratory analyzed the function of SUSD2 in breast tumorigenesis.^15^ Using *in vitro* phenotypic assays, we showed that overexpression of *SUSD2* in MDA-MB-231 cells increased invasion and contributed to an immune evasion mechanism through induction of apoptosis of T cells.^15^ Furthermore, using a syngeneic mouse model, we revealed that mice with *SUSD2*-expressing tumors had poorer survival and increased tumor growth rates compared with mice with tumors not expressing *SUSD2*.^15^

Currently, SUSD2 has not been studied in the context of EOC. Surprisingly, immunohistochemical (IHC) analysis revealed that HGSOC patients with high levels of SUSD2 staining in their primary ovarian tumors have a better prognosis compared with patients with low levels of SUSD2 staining. Unlike the oncogenic function of SUSD2 in breast cancer, this result suggests that SUSD2 may function by attenuating tumorigenesis in HGSOCs. Because SUSD2 has several domains implicated in cell–cell adhesion, we hypothesized that SUSD2 inhibits processes in HGSOCs that depend on the loss of cell–cell interaction, such as migration, EMT and mesothelial clearance.

## Results

### IHC analysis of SUSD2 in ovarian cancer and normal tissues

**Figure 1:**
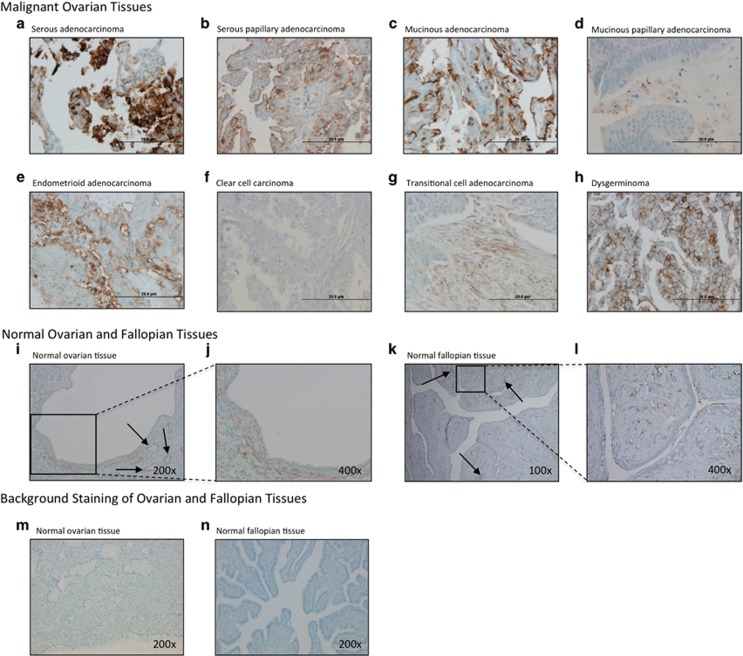
IHC staining of SUSD2 in ovarian tissues. Human ovarian tissues (obtained from a BioMax TMA) and paraffin-embedded sections of normal ovary and fallopian tube were stained by IHC analysis using an anti-SUSD2 antibody and counterstained with hematoxylin. The brown color indicates the presence of SUSD2. Ovarian tissues were imaged at a magnification of × 200. Scale bars indicate 20 μm. (**a**) Grade 3, stage I, serous adenocarcinoma. (**b**) Grade 2, stage IIIc, serous papillary adenocarcinoma. (**c**) Grade 2, stage Ib, mucinous adenocarcinoma. (**d**) Grade 1, stage I mucinous papillary adenocarcinoma. (**e**) Grade 2, stage Ia endometrioid adenocarcinoma. (**f**) Stage I clear-cell carcinoma. (**g**) Grade 2, stage Ia transitional cell adenocarcinoma. (**h**) Stage Ib dysgerminosa. (**i**) Normal ovarian tissue imaged at a magnification of x100. (**j**) Increased magnification (× 400) of area in black box of (**i**). (**k**) Normal fallopian tissue imaged at a magnification of × 100. (**l**) Increased magnification (× 400) of area shown in black box of (**k**). (**m**, **n**) Normal ovarian and fallopian tissues stained with secondary antibody only (imaged at a magnification of × 200).

To determine the presence of SUSD2 in ovarian tissue, we performed IHC analysis using an anti-SUSD2 antibody on a US BioMax human ovarian tissue microarray (TMA). The TMA contained a total of 208 patient samples, 192 of which were primary tumors representing several subtypes of ovarian carcinomas including serous, mucinous, endometrioid, clear cell, transitional cell and dysgerminoma. The remaining 16 samples were benign ovarian tissue. Because the sizes of the cores on the tissue array are limited, a paraffin-embedded cross-section of a benign ovary was also stained. Representative images of SUSD2 staining in malignant and benign ovarian tissues are shown in Figures 1a–j.

With the exception of clear-cell carcinoma, patient tissue samples of dysgerminoma and all subtypes of ovarian adenocarcinomas displayed positive SUSD2 epithelial cell staining (Figures 1a–e and f). Although staining intensity varied between subtypes, tumors of patients with HGSOC consistently displayed high levels of SUSD2 staining (Figure 1a). Because HGSOC originates from the fallopian tube,^16, 17, 18^ normal fallopian tissue was also stained for the presence of SUSD2. Consistent with our previous findings regarding normal breast tissue,^15^ normal ovarian tissue displayed weak, diffuse SUSD2 staining in the epithelial cells with moderate staining in the endothelial lining of vessels (Figures 1i and j). Similarly, normal fallopian epithelial cells displayed weak, diffuse SUSD2 staining with slightly increased intensity of staining in the vascular endothelial cells (Figures 1k and l). Images of normal fallopian and ovarian tissues stained with secondary antibody only displayed no positive staining, validating the specificity of binding between the primary antibody and SUSD2 (Figures 1m and n).

### IHC analysis reveals that increased patient survival correlates with high levels of SUSD2 staining in HGSOC tumors

**Figure 2:**
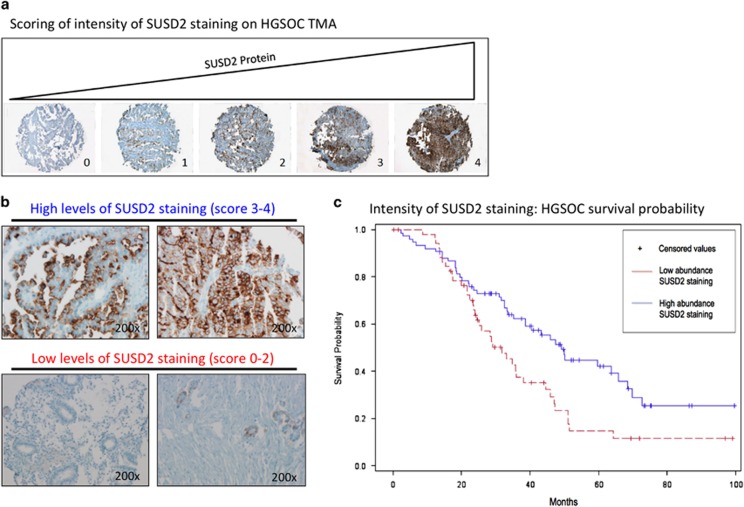
Increased patient survival correlates with strong SUSD2 IHC staining in HGSOCs. A TMA containing 128 biopsies from patients diagnosed with HGSOCs was analyzed by immunohistochemistry using an antibody against SUSD2. The brown color indicates the presence of SUSD2, and the slides were counterstained with hematoxylin, which stains the nucleus blue. A pathologist scored the stained TMA. (**a**) Representative photos of the five categories of SUSD2 staining of the HGSOC TMA. Percentage of SUSD2 staining was scored from 0 to 4: 0 representing no SUSD2 stained cells and 4 representing >75% of cells stained for SUSD2. (**b**) IHC staining of SUSD2 in HGSOC. In the top panel, images of HGSOC tissues representing extensive SUSD2 staining are shown. In the bottom panel, images of HGSOC tissues representing a low level of SUSD2 staining are shown. (**c**) Kaplan–Meier curve showing patient survival correlated to SUSD2 staining. The red line represents low abundance of SUSD2, and the blue line indicates high abundance of SUSD2 staining. Patients with low levels of SUSD2 staining survived an average of 31.7 months after initial diagnosis. Patients with high levels of SUSD2 staining survived an average of 49.1 months after initial diagnosis. *P*-values were calculated using log-rank values, which weight values at the end of the curve more heavily, and Wilcoxon's transformed values, which weight value at the beginning of the curve more heavily; *P*=0.0083 and 0.0320, respectively.

Results from the US BioMax human ovarian TMA indicated that SUSD2 levels were highest in the HGSOC subtype (Figure 1). HGSOC is the most commonly diagnosed and is considered the deadliest of ovarian carcinoma subtypes with <40% of HGSOC patients surviving 5 years after initial diagnosis.^3^ To investigate the effect of SUSD2 on overall survival of HGSOC patients, a clinically annotated TMA containing 128 primary tumors from patients diagnosed with HGSOC was analyzed by IHC using an anti-SUSD2 antibody (Figures 2a–c). For each patient sample, a trained pathologist scored the percentage of cancer epithelial cells that stained positive for SUSD2 on the HGSOC TMA (see Materials and methods). Roughly 60% of the HGSOC tumors on the TMA had high levels of SUSD2 staining (Figure 2b), and ~40% displayed low levels (Figure 2c). The level of SUSD2 staining was then correlated to patient survival, generating a Kaplan–Meier curve (Figure 2d). HGSOC patient samples with high levels of SUSD2 staining survived an average of 18 months longer compared with HGSOC patient samples with low SUSD2 staining (median survival of 31.7 and 49.1 months, respectively; *P*-value=0.0083), suggesting that high SUSD2 levels in HGSOC tumors are associated with a better patient prognosis (Figure 2c). No statistically significant correlation between the extensiveness of SUSD2 staining, platinum sensitivity, cytoreduction or stage (Federation of Gynecology and Obstetrics) of HGSOC progression was observed among the tissue samples studied.

### Generation and characterization of SUSD2-knockdown HGSOC cell lines

**Figure 3:**
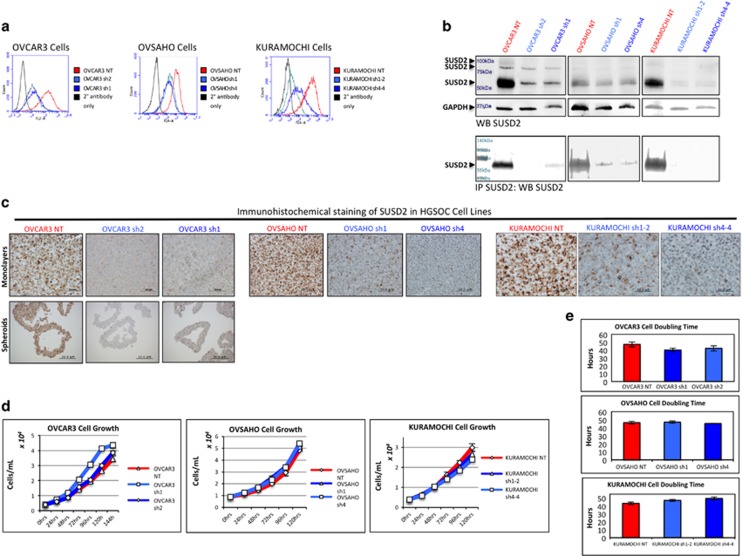
Characterization of NT and *SUSD2*-KD HGSOC cell lines. (**a**) Flow cytometry analysis. Flow cytometry analysis was performed on HGSOC cells (OVCAR3, OVSAHO and KURAMOCHI cell lines) using an anti-SUSD2 antibody. All HGSOC cell lines shown were transfected with either an NT shRNA construct (abbreviated ‘NT' shown in red) or one of two *SUSD2*-targetting shRNA constructs (abbreviated ‘sh1/2/4/1-2/4-4' shown in shades of blue). The black curves in each graph depicts the background fluorescence observed when nonspecific binding of the secondary antibody alone binds to cell surface proteins. Fluorescence is shown in the x axis and the number of cells is shown in the y axis. (**b**) Analysis of SUSD2 levels in HGSOC whole-cell lysates. The upper two panels show protein bands from western (WB) immunoblot analysis detecting SUSD2 and GAPDH in whole-cell lysates. In the lower panel, immunoprecipitation (IP) reactions on HGSOC whole-cell lysates were performed using an anti-SUSD2 antibody. Immunoprecipitated proteins were separated on a sodium dodecyl sulfate polyacrylamide gel electrophoresis (SDS–PAGE) gel followed by WB analysis using an anti-SUSD2 antibody. The band representing full-length, post-translationally modified SUSD2 is ~100 kDa. The 90 kDa band represents partially glycosylated SUSD2 protein, and the strongest band represents a post-translationally cleaved ~60 kDa SUSD2 product. (**c**) IHC analysis of SUSD2 in HGSOC cells/spheroids. HGSOC cell lines and OVCAR3 spheroids were pelleted into buttons before being formalin fixed and embedded in paraffin. OVCAR3 spheroids were formed in 24 h by plating OVCAR3 cells in ultra-low attachment plates (20 000 cells/spheroid). Using an anti-SUSD2 antibody, IHC analysis was performed on HGSOC cell line monolayers and OVCAR3 spheroids. Brown indicates positive SUSD2 staining, and blue indicates cell nuclei (counterstained with hematoxylin). Representative images are shown imaged at x200 magnification. (**d**, **e**) Cell proliferation analysis. HGSOC cell lines were plated in triplicate in selection media and counted using a Coulter counter every 24 h for 6 days (144 h) to assess cell growth. (**e**) HGSOC cell counts over the course of linear growth were used to calculate cell-doubling time.

To investigate the biological consequences of *SUSD2* expression, we used three HGSOC cell lines (OVCAR3, OVSAHO and KURAMOCHI), all of which have been determined to contain a p53 mutation as well as several substantial copy-number changes associated with HGSOC.^19^ OVCAR3, OVSAHO and KURAMOCHI cells endogenously express *SUSD2*, and the encoded protein is localized on the cell surface. To study the function of SUSD2, a non-targeting (NT) control cell line and two SUSD2-knockdown (KD) stable cell lines were generated for OVCAR3, OVSAHO and KURAMOCHI cells using short hairpin RNA (shRNA) technology (referred to as OVCAR3 NT/sh1/sh2, OVSAHO NT/sh1/sh4 and KURAMOCHI NT/sh1-2/sh4-4, respectively). Flow cytometry analysis confirmed that the KD cell lines contained very low levels of SUSD2 compared with the NT control (Figure 3a). SUSD2 protein levels were examined by western immunoblot analysis using whole-cell lysates harvested from OVCAR3, OVSAHO and KURAMOCHI stable cell lines (Figure 3b).

SUSD2 is composed of 822 amino acids with a predicted molecular weight of 90.4 kDa. Because SUSD2 is glycosylated and post-translationally cleaved, three bands are detected when performing western immunoblot analysis on whole-cell lysates: a faint ~100 kDa band, representing the glycosylated, full-length protein, a weak ~90 kDa band, representing full-length SUSD2 with minimal glycosylation, and a strong ~60 kDa band, representing a cleaved product of SUSD2 (Figure 3b, top panel).^20^ In addition, immunoprecipitation followed by western immunoblot analysis was performed using whole-cell lysates harvested from OVCAR3, OVSAHO and KURAMOCHI stable cell lines (Figure 3b, bottom panel). The prominent ~60 kDa band is much weaker in the stable SUSD2-KD HGSOC cell lines as compared with the NT controls (Figure 3b, bottom panel). OVCAR3, OVSAHO and KURAMOCHI NT and KD cell lines were grown as monolayers. In addition, OVCAR3 NT/sh1/sh2 cells were grown as spheroids. All cell lines were harvested, pelleted and paraffin embedded. IHC analysis was performed on sections using an anti-SUSD2 antibody. Consistent with the results from flow cytometry and western immunoblot analysis, the NT control cell lines showed strong SUSD2 membrane staining, whereas weak to no staining was observed in the HGSOC SUSD2-KD cell lines (Figure 3c).

To examine whether SUSD2 affects the growth of HGSOC cells, cell proliferation was assayed by counting cells using a Coulter counter over the course of 5 to 6 days (120–144 h; Figure 3d). Cell-doubling times were extrapolated from the growth curves of SUSD2-KD and NT HGSOC cell lines, and no significant difference in cell growth rates was observed in the first 48 h (Figure 3e).

### Decreased levels of SUSD2 on HGSOC cells increase migration

**Figure 4:**
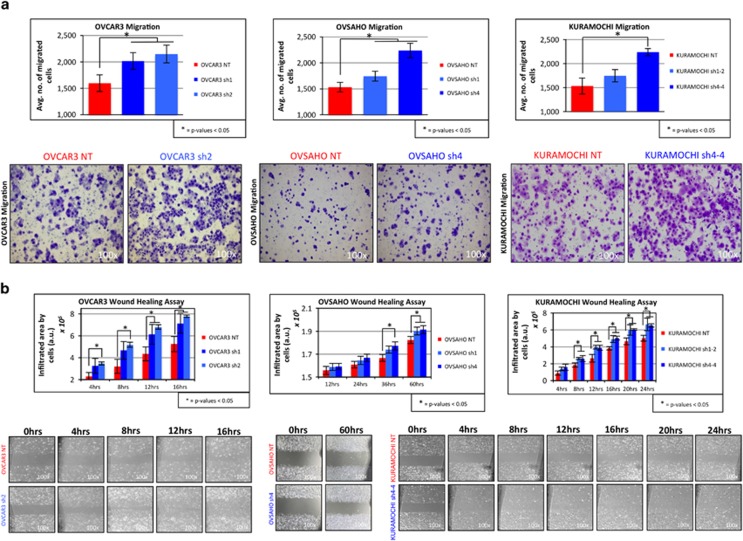
Decreased SUSD2 levels increased migration of HGSOC cells. (**a**) Boyden chamber analysis. Uncoated Boyden chambers were used to analyze migration of HGSOC cells (OVCAR3, OVSAHO and KURAMOCHI cell lines). Cells that migrated through the chambers were stained with crystal violet and quantitated, as represented in the bar graphs in the top panel. Images of stained OVCAR3 and KURAMOCHI cells were taken 24 h after seeding the experiment. Images of stained OVSAHO cells were taken 48 h after seeding the experiment. Representative images of the experimental outcomes from both the NT and most efficient SUSD2-KD HGSOC cell line model are displayed in the lower panel (images were viewed with a × 10 objective using bright field light microscopy). All migration experiments were performed in triplicate. (**b**) Wound-healing analysis. Migration of HGSOC cell lines was investigated using a wound-healing assay. The graphs in the top panel show the total area occupied by the designated HGSOC cell line at different time points throughout the experiment. Representative images are displayed in the lower panel of the figure (images were viewed with a × 10 objective using bright field light microscopy). The area occupied by the invading HGSOC cells was calculated using arbitrary units (a.u.) derived from the ImageJ software analysis. All experiments were performed in triplicate. *P*-values were derived using a standard two-tailed *t*-test between SUSD2-KD cells and NT controls.

Boyden chamber assays were performed to determine whether SUSD2 influences the migration of HGSOC cells. OVCAR3 NT/sh1/sh2, OVSAHO NT/sh1/sh4 and KURAMOCHI NT/sh1-2/sh4-4 cell lines were plated on the membranes of the culture inserts and allowed to migrate toward a chemoattractant in the lower chamber (10% fetal bovine serum). As shown in Figure 4a (top panel), with the exception of KURAMOCHI sh1-2 cell line, the SUSD2-KD cell lines (OVCAR3 sh1/sh2, OVSAHO sh1/sh4 and KURAMOCHI sh4-4) showed a significantly greater rate of migration through the membrane when compared with their complimentary NT cell lines (OVCAR3 NT, OVSAHO NT and KURAMOCHI NT, respectively), suggesting that SUSD2 inhibits cellular migration in HGSOC. Boyden chamber invasion assays were performed using Matrigel, and no significant differences were observed between the NT and SUSD2-KD cell lines (data not shown).

Migration was also investigated using an artificial wound-healing assay using OVCAR3 NT/sh1/sh2, OVSAHO NT/sh1/sh4 and KURAMOCHI NT/sh1-2/sh4-4 cell lines (Figure 4b). Quantification of cells infiltrating the artificial wound revealed that the SUSD2-KD cell lines (OVCAR3 sh1/sh2, OVSAHO sh1/sh4 and KURAMOCHI sh1-2/sh4-4) closed the artificial wound at significantly higher rates compared with their complimentary NT cell lines (OVCAR3 NT, OVSAHO NT and KURAMOCHI NT, respectively; Figure 4b). In general, OVSAHO cells migrated at a much slower rate than the OVCAR3 and KURAMOCHI cells, as 60 h were required for the assay, compared with 16 or 24 h, respectively (Figure 4b). These results demonstrate that decreased SUSD2 levels in HGSOC cells increased cell migration, verifying our results obtained from the Boyden chamber migration assay.

### Decreased SUSD2 in OVCAR3, OVSAHO and KURAMOCHI cells increased mRNA and protein associated with EMT

**Figure 5:**
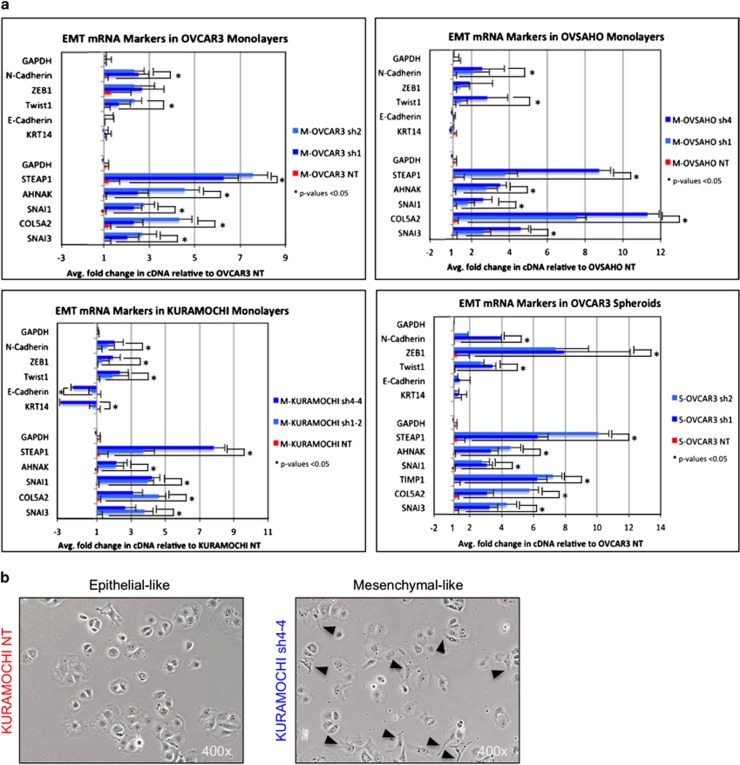
Decreased SUSD2 levels increased mRNA associated with EMT. (**a**) RT-qPCR **a**nalysis of EMT mRNA markers in HGSOC cells (OVCAR3, OVSAHO and KURAMOCHI cell lines). RNA was isolated from HGSOC cell line monolayers and OVCAR3 spheroids, and the generated cDNA was used as a template for RT-qPCR. The graphs depict two separate experiments (indicated by the separation of genes in the y axis) investigating the effect of SUSD2 levels on mRNA expression of several well-characterized EMT genes (*N-cadherin*, *ZEB1*, *Twist1*, *E-Cadherin KRT14*,*STEAP1*, *AHNAK*, *SNAI1*, *COL5A2* and *SNAI3*). Fold changes were calculated by comparing the levels of mRNA expression observed in KD cell lines to NT cell lines (Ct values were calculated via the comparative Ct method). Each experiment was performed in triplicate. *P*-values were derived using a standard two-tailed *t*-test between SUSD2-KD cells and NT controls. (**b**) Morphology of KURAMOCHI cells. Macroscopic images of adherent KURAMOCHI cells were taken using bright field light microscopy via a high-powered objective (× 400 magnification). KURAMOCHI NT cells are pictured on the left (cells expressing endogenous levels of SUSD2 protein) and KURAMOCHI sh4-4 cells are pictured on the right (cells stably transfected with functional *SUSD2*-targeting shRNA). Black arrows indicate elongations from filopodia formation evident in the KURAMOCHI sh4-4 cells.

Because increased migration is often correlated with EMT, we investigated the potential role of SUSD2 in EMT regulation. RT-qPCR analysis was performed on RNA isolated from OVCAR3 NT/sh1/sh2, OVSAHO NT/sh1/sh4 and KURAMOCHI NT/sh1-2/sh4-4 monolayers and OVCAR3 NT/sh1/sh2 spheroid cultures. The derived cDNA was amplified using primers specific to two genes associated with an epithelial phenotype, *CDH1* (*E-cadherin)* and *Keratin 14*, and eight genes associated with a mesenchymal phenotype, *CDH2* (*N-cadherin)*, *ZEB1*, *TWIST1 (Twist-1)*, *SNAI3 (Snail-3)*, *COL5A2 (Collagen, Type V, Alpha 2)*, *SNAI1 (Snail-1)*, *AHNAK (AHNAK Nucleoprotein)*and *STEAP1 (Six Transmembrane epithelial antigen of the Prostate 1)*. Fold changes in mRNA levels were calculated based on Ct values acquired from the RT-qPCR analysis (Figure 5a). Compared with the OVCAR3 NT, OVSAHO NT, and KURAMOCHI NT monolayer cell lines, the corresponding monolayer SUSD2-KD cell lines (OVCAR3 sh1/sh2, OVSAHO sh1/sh4 and KURAMOCHI sh1-2/sh4-4 cell lines) had significantly greater mRNA expression of mesenchymal genes, *STEAP1*, *AHNAK*, *Snail-1*, *COL5A2* and *Snail-3.* Furthermore, with the exception of KURAMOCHI sh4-4, these SUSD-KD cell lines showed no statistical differences in epithelial mRNA expression of *E-cadherin* or *Keratin-14* relative to the NT cell lines (OVCAR3 NT, OVSAHO NT and KURAMOCHI NT). Moreover, for the majority of the mesenchymal genes assayed, the clones with the more efficient SUSD2-KD (OVCAR3 sh2, OVSAHO sh4 and KURAMOCHI sh4-4) showed a greater mRNA expression value when compared with their partial SUSD2-KD counterpart (OVCAR3 sh1, OVSAHO sh1 and KURAMOCHI sh1-2 cell lines), suggesting that the amount of upregulation of mesenchymal genes is dependent of the levels of SUSD2 in HGSOC cells (Figure 5a). Similar upregulation of mesenchymal mRNA in SUSD2-KD cells was observed in OVCAR3 cells grown as spheroids (Figure 5a). No significant differences in expression of *E-cadherin* and *Keratin-14* were observed between OVCAR3 NT/sh1/sh2 spheroids (Figure 5a). Interestingly, KURAMOCHI sh4-4 cells represented the only cell line to show significant downregulation of epithelial genes, *E-cadherin* and *Keratin-14*, when compared with its complementary NT cell line, KURAMOCHI NT, −2.2- and −2.8 fold, respectively (Figure 5a).

To investigate whether changes in expression of well-characterized EMT genes affected the morphology of the HGSOC cell lines, bright field microscopy images were taken of adherent HGSOC cells grown as monolayers. Examining the images revealed that the SUSD2-KD cell lines (OVCAR3 sh1/sh2, OVSAHO sh1/sh4 and KURAMOCHI sh1-2/sh4-4) shared subtle changes in morphology, including a more elongated shape, when compared with their complimentary NT cell lines (OVCAR3 NT, OVSAHO NT and KURAMOCHI NT). These morphological changes were most notable in comparing KURAMOCHI NT cells to KURAMOCHI sh4-4 cells as shown in Figure 5b. Altogether, RT-qPCR and morphological investigation would suggest that SUSD2 is associated with a more epithelial-like phenotype in HGSOC cells.

**Figure 6:**
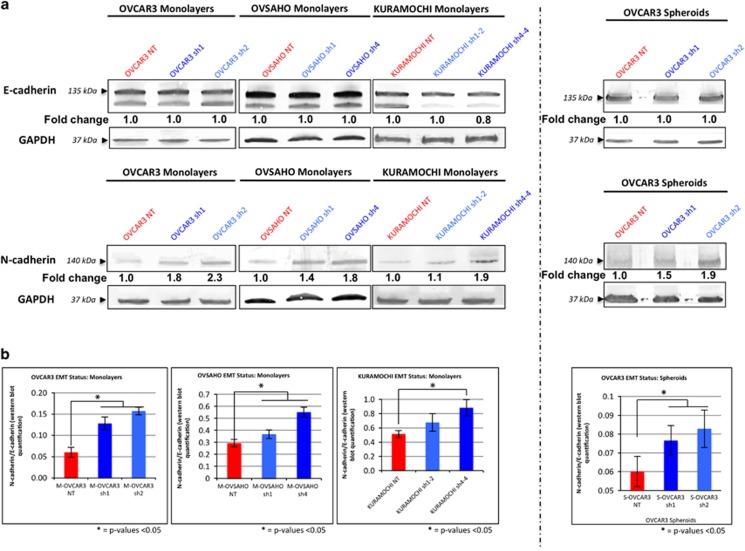
Decreased SUSD2 levels increased protein expression of N-cadherin. (**a**) Western immunoblot analysis of N- and E-cadherin proteins in HGSOC cells (OVCAR3, OVSAHO and KURAMOCHI cell lines). Proteins from whole-cell lysate were harvested from HGSOC cell line monolayers and OVCAR3 spheroids. The bold numbers shown below the E- and N-cadherin bands represent the fold change in the protein level of E- and N-cadherin, respectively, relative to the NT HGSOC cell line assayed. Fold changes in OVCAR3, OVSAHO and KURAMOCHI cell lines are relative to protein levels in the corresponding NT cell line. (**b**) Quantification of N-cadherin/E-cadherin in HGSOC cell lines. The bar graphs depict the ratio of N-cadherin/E-cadherin detected in western immunoblot analysis. When calculating protein values of N-/E-, N- and E-cadherin expression were initially normalized to GAPDH protein levels. *P*-values were derived using a standard two-tailed *t*-test between the SUSD2-KD protein lysates (monolayers and spheroids as indicated) and corresponding NT lysate values.

Western immunoblot analysis of E- and N-cadherin using whole-cell lysates derived from OVCAR3 NT/sh1/sh2, OVSAHO NT/sh1/sh4 and KURAMOCHI NT/sh1-2/sh4-4 cell line monolayers as well as OVCAR3 NT/sh1/sh2 spheroid cultures (Figure 6a) was performed. The subsequent results reflected the previously described RT-qPCR results, revealing that E-cadherin protein levels were unaffected by SUSD2 levels in OVCAR3 and OVSAHO cells (including the OVCAR3 cells grown as spheroids). Consistently, downregulation of E-cadherin was observed in KURAMOCHI SUSD2-KD cell lines (KURAMOCHI sh1-2 and KURAMOCHI sh4-4) when compared with KURAMOCHI NT cells (Figure 6a). Similarly, the amount of N-cadherin in the OVCAR3, OVSAHO and KURAMOCHI SUSD2-KD cell lines was increased when compared with their complimentary NT cell lines (ranging from a 1.1-fold increase observed in KURAMOCHI sh1-2 monolayers to a 2.3-fold increase observed in OVCAR3 sh2 monolayers; Figure 6a).

These data indicate an inverse correlation of SUSD2 with the mesenchymal protein, N-cadherin, such that when OVCAR3 cells have low levels of SUSD2, levels of N-cadherin are increased. To demonstrate this correlation and to provide a simplistic measurement of EMT status as it relates to SUSD2 in HGSOC cells, we calculated a ratio of N-cadherin/E-cadherin in all HGSOC cell line monolayers and spheroids (Figure 6b). OVCAR3 sh1/sh2, OVSAHO sh1/sh4 and KURAMOCHI sh4-4 monolayers as well as OVCAR3 sh1/sh2 spheroids were found to have significantly higher N-cadherin/E-cadherin ratios compared with their complimentary NT cell lines (OVCAR3 NT, OVSAHO NT and KURAMOCHI NT monolayers, and OVCAR3 NT spheroids, respectively; Figure 6b). Although there was no significant increase in N-cadherin/E-cadherin observed between KURAMOCHI sh1-2 and KURAMOCHI NT cell lines, a similar trend was evident that paralleled the results of the remaining SUSD2-KD cell lines and their complimentary NT cell lines, suggesting that the presence of SUSD2 positively correlates with a more epithelial-like phenotype.

### Decreased levels of SUSD2 in OVCAR3 spheroids increase mesothelial clearance

**Figure 7:**
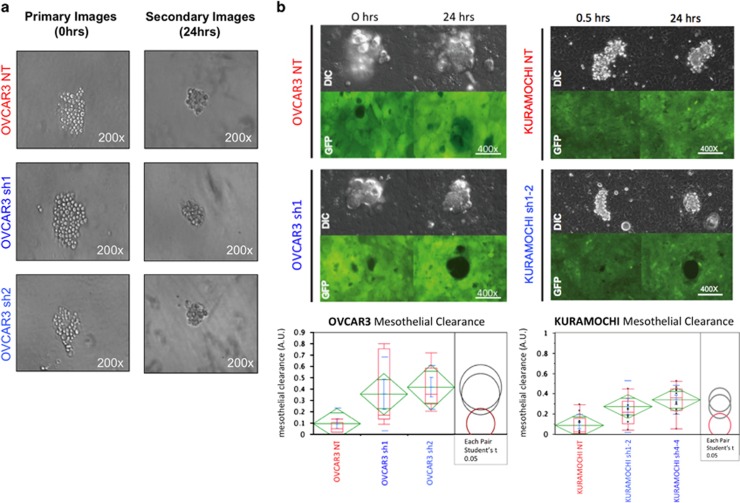
OVCAR3 and KURAMOCHI spheroids with KD levels of SUSD2 correlated with increased mesothelial clearance. (**a**) Images of spheroid formation. OVCAR3 and KURAMOCHI cells were plated in poly-HEMA-coated 96-well round bottom plates for spheroid formation (~100 cells per spheroid). Representative images of OVCAR3 cells were taken at 0 and 24 h after plating. (**b**) Representative images of spheroid–mesothelial interaction. OVCAR3 and KURAMOCHI cell lines were induced to form spheroids by plating them in ultra-low attachment plates for 24 h. The spheroids were then plated atop a human monolayer of mesothelial cells expressing GFP. DIC images show spheroid interacting with the mesothelium at times 0/0.5 h and 24 h. GFP images (bottom) show changes made by spheroids to the integrity of the mesothelium at times 0 and 24 h. Quantification of mesothelial clearance by spheroids is depicted in the graph located below the images. All experiments were performed in triplicate. *P*-values were derived using a standard two-tailed *t*-test between the OVCAR3 sh1/sh2 cells and OVCAR3 NT values or the KURAMOCHI sh1-2/sh4-4 cells and KURAMOCHI NT values as indicated.

The complex process of spheroid implantation involves integrin/talin-dependent activation of myosin to produce traction force to displace mesothelial cells from underneath a tumor spheroid.^9^ To determine if SUSD2 affects spheroid implantation in HGSOC, we performed *in vitro* mesothelial clearance assays using OVCAR3, OVSAHO and KURAMOCHI stable cell lines. Spheroids were placed directly on a confluent monolayer of green florescence protein (GFP) expressing mesothelial cells (Figure 7b). Live-cell microscopy revealed that the OVCAR3 NT and KURAMOCHI NT spheroids cleared significantly fewer mesothelial cells compared to the clearance achieved by the OVCAR3 and KURAMOCHI SUSD2-KD spheroids (Figure 7b; *P*-value>0.05). These data suggest that SUSD2 impedes the ability of OVCAR3 and KURAMOCHI spheroids to breach the mesothelium. No significant increase in mesothelial clearance of the *GFP*-expressing mesothelial layer was observed with the OVSAHO SUSD2-KD spheroids compared with the NT control (data not shown). However, in the aforementioned mesothelial clearance assay, clearance is measured after a 24-h incubation. The assays performed when using OVSAHO spheroids suggest that these spheroids may require a longer period of time to clear the mesothelial layer, which does not fall within the parameters of this assay.

## Discussion

The majority of HGSOC patients are diagnosed at a late stage of disease progression when treatment options are limited and poor in efficacy,^3^ concurrent with a 5-year survival rate of 17–39%.^4^ Our results indicated that HGSOC patients with high levels of SUSD2 staining in their primary tumors survive an average of 18 months longer than those with low levels of SUSD2 staining (Wilcoxon's transformed *P*-value=0.032; Figure 2c), revealing a significant increase in survival among HGSOC patients with late-stage diagnosis. Analysis of HGSOC sample sets from The Cancer Genome Atlas showed support for a relationship between *SUSD2* copy-number and overall survival in HGSOC tumors, defined by an overall increase in survival in patients with an amplified copy number of *SUSD2* alleles (data not shown). However, because of the small number of samples, statistical significance could not be attained. Using the same HGSOC sample sets, no significant correlation between *SUSD2* mRNA levels and patient survival was observed (data not shown). Because protein data was not available for these patient samples, it is unclear whether protein levels corresponded directly with *SUSD2* expression.

Cancer cells possess a broad spectrum of migration and invasion mechanisms that include both individual and collective cell-migration strategies.^21, 22^ SUSD2 contains several domains frequently found in molecules implicated in cell–cell and cell–matrix adhesion,^15^ which suggests a potential role in the regulation of cell migration and/or invasion. Here, we demonstrated that KD levels of SUSD2 increased migration of OVCAR3, OVSAHO and KURAMOCHI cells through an artificial basement membrane and into an artificial wound (Figures 4a and b), indicating that SUSD2 may impede cell migration in HGSOC. Cell motility, often characterized by the migration potential of cells, represents a key feature associated with EMT, a process characterized by altered signaling pathways in epithelial cells. EMT induces cellular changes in expression profiles defined by downregulation of epithelial genes and upregulation of mesenchymal genes,^23^ resulting in the loss of cell–cell adhesion and apical–basal polarity, inducing cell motility, invasiveness, increased resistance to apoptosis and often leading to poorer patient prognosis.^23, 24^ It has been proposed that EMT-induced cell motility represents an important step in the exfoliation of epithelial cells from the surface of the primary tumor into the peritoneal cavity in passive dissemination of EOC.^11, 12, 25^ In the present study, RT-qPCR analysis revealed that decreased *SUSD2* expression in OVCAR3, OVSAHO and KURAMOCHI monolayers and OVCAR3 spheroids correlated with the upregulation of mRNA encoding for several well-characterized mesenchymal proteins (N-cadherin, Twist-1, ZEB1, STEAP1, AHNAK, Snail-1, COL5A2 and Snail-3; Figure 5a).

A common predictor of EMT is the ‘cadherin switch', defined by reduced expression of E-cadherin and increased expression of N-cadherin.^26, 27, 28^ In a past study, Quattrocchi *et al.*^29^ examined cadherin expression in 177 HGSOC paraffin-embedded patient tumors, revealing a significant inverse correlation between N-cadherin and overall survival. Here, we demonstrated that the protein ratio of N-cadherin/E-cadherin increased when *SUSD2* expression was knocked down in OVCAR3, OVSAHO and KURAMOCHI monolayers and OVCAR3 spheroids (Figures 6a and b). This mRNA and protein analysis of EMT markers suggests that expression of *SUSD2* may correlate with a more epithelial phenotype in HGSOC cells, driven specifically by the inverse relationship between levels of SUSD2 and N-cadherin. These findings implicate SUSD2 as a mediator of tumor suppression in HGSOC through the attenuation of EMT-induced cell migration, a potential mechanism for reduction in exfoliation of the primary tumor in HGSOC progression.

It is worth noting that reduced expression of *SUSD2* in OVCAR3 and OVSAHO cells as well as in OVCAR3 spheroids did not significantly affect gene expression of *Keratin-14* and *E-cadherin* or protein levels of E-cadherin (Figures 5a and 6a). Keratin-14 and E-cadherin are associated with an epithelial phenotype in many epithelial cancers; however, some traditionally classified EMT markers, such as Keratin-14 and E-cadherin, have yet to be fully understood in the context of HGSOC.^30^ In a previous study, it was shown that ovarian cancer cells expressed both epithelial and mesenchymal markers during an intermediate state of EMT.^31^ In a more recent publication, Carduner *et al.*^33^ showed that intermediate EMT phenotypes have the potential to upregulate mesenchymal markers with E-cadherin protein levels unaltered. Siversten *et al.*^32^ demonstrated that malignant ovarian effusions contained both N-cadherin and E-cadherin. Therefore, it is not unexpected to observe mesenchymal characteristics that are induced by KD of *SUSD2* with unaltered levels of Keratin-14 and E-cadherin.

In EOC, metastatic dissemination depends on the ability of spheroids to implant onto the peritoneal wall. Successful spheroid implantation requires the displacement of the peritoneal mesothelium, a thin monolayer composed of mesothelial cells covering the peritoneal cavity.^34^ In a recent publication, Davidowitz *et al.*^35^ provided *in vitro* assessment of mesothelial clearance in 20 established EOC cell lines. Examination of gene and protein expression profiles of the 20 established EOC cell lines suggested that mesothelial clearance by spheroids depends on their EMT status. This study demonstrated that spheroids composed of mesenchymal-like cells were able to clear mesothelial cells significantly better than those composed of epithelial-like cells. Our results from the mesothelial clearance assay showed that reduced levels of SUSD2 in OVCAR3 and KURAMOCHI spheroids correlated with increased mesothelial clearance (Figure 7b). OVCAR3 cells have previously been shown to be a highly efficient mesothelial-clearance competent cell line.^35^ By decreasing the levels of SUSD2, we generated OVCAR3 cell lines (OVCAR3 sh1 and sh2) with an exaggerated clearance phenotype or a ‘super-clearance' cell line. This super-clearance phenotype may be a result of enhanced EMT progression as demonstrated by the increased N-cadherin/E-cadherin protein ratio observed in the SUSD2-KD cell lines.

The results from this study suggest that SUSD2 has tumor-suppressive properties in HGSOC, whereas the results from our previous study investigating the role of SUSD2 in breast cancer suggested that SUSD2 increases tumorigenesis, including angiogenesis.^15^ Although initially surprising, the contrasting phenotypes of SUSD2 in breast and ovarian cancers could be explained by differences in metastasis mechanisms. Because HGSOC does not require angiogenesis as a means of metastasis,^6, 7^ whereas breast cancer almost exclusively relies on angiogenesis to metastasize,^36, 37^ the role of SUSD2 as observed in breast cancer may not manifest any obvious advantage in dissemination in HGSOC. Future studies investigating how *SUSD2* expression in HGSOC may impact other factors that affect patient outcome, such as chemotherapy resistance of spheroids or the ability of the cancer cells to evade the immune system, will increase our understanding of the dynamic phenotypes of SUSD2 in both breast and ovarian cancers. In addition, determining the protective function of SUSD2 in HGSOC tumors may uncover useful knowledge in the development of novel therapies to improve survival of HGSOC patients.

## Materials and methods

### Cell culture

OVCAR3 cell lines were purchased from ATCC (Manassas, VA, USA) and maintained in Dulbecco's modified Eagle's medium with 10% fetal bovine serum (Atlanta Biologicals, Flowery Branch, GA, USA), 1 μg/ml puromycin and grown at 37 °C in humidified 5% CO_2_. OVSAHO and KURAMOCHI cell lines were a generous gift from the Drapkin Laboratory (Philadelphia, PA, USA). OVSAHO cell lines were maintained in RPMI with 10% fetal bovine serum (Atlanta Biologicals), 1% penicillin–streptomycin (HyClone, Pittsburgh, PA, USA), 1 μg/ml puromycin and grown at 37 °C in humidified 5% CO_2_. KURAMOCHI cell lines were maintained in RPMI with 10% fetal bovine serum, 0.25 U/ml of human insulin (Sigma-Aldrich Corp., St Louis, MO, USA), 1 × MEM (1 ml amino acids/100 ml of media) of non-essential amino acids (HyClone), 1 μg/ml puromycin and grown at 37 °C in humidified 5% CO_2_. Cell lines were authenticated by STR profiling (IDEXX BioResearch, Columbia, MO, USA). All cell lines test negative for mycoplasma.

### Construction of stable cell lines

OVCAR3, OVSAHO and KURAMOCHI cell lines endogenously produce high levels of SUSD2. *SUSD2* shRNA-expressing lentiviral particles (pLKO.1 vector, MISSION shRNAs; Sigma-Aldrich Corp.) were used to generate stable SUSD2-KD cell lines. An NT shRNA sequence was used as a control (sequences: SUSD2 sh1, 5′-CCGGGACGATCATTTCTGCAACTTTCTCGAGAAAGTTGCAGAAATGATCGTCTTTTTTG-3′; SUSD2 sh2, 5′-CCGGCATCTACTTCCACTGTGACAACTCGAGTTGTCACAGTGGAAGTAGATGTTTTTTG-3′ SUSD2 sh4, 5′-CCGGCCAAATACTCACGGCTCTAATCTCGAGATTAGAGCCGTGAGTATTTGGTTTTTTG-3′; and NT control, 5′-CCGGGACGATCATTTCTGCAACTTTCTCGAGAAAGTTGCAGAAATGATCGTCTTTTTTG-3′). Cells were infected according to the manufacturer's instructions and selected with 1 μg/ml puromycin. Stable clones were selected for further study based on the extent of SUSD2-KD determined by flow cytometry. To further enrich the OVCAR3 cell population for low SUSD2 levels, a FACSJazz fluorescence-activated cell sorter was used.

### Flow cytometry

All HGSOC cells lines were assessed for cell-surface levels of SUSD2 as described previously.^15^ HGSOC cells were stained with a rat monoclonal anti-SUSD2 antibody followed by a fluorescently conjugated secondary antibody. Samples were analyzed using a Accuri Flow Cytometer (San Diego, CA, USA) and gated based on forward- and side-scatter profiles to exclude debris and cellular aggregates from the analysis. Three biological replicates were performed for all flow cytometry experiments. The rat monoclonal anti-SUSD2 antibody was generated by Aldevron (Freiburg, Germany) as a custom order for the Egland Lab (Sioux Falls, SD, USA). The Egland Lab provided the *SUSD2* full-length expression construct (based on NCBI Reference Sequence: NM_019601.3) for DNA immunization of rats. Hybridomas were generated, and clones producing secreted antibodies that recognized the native SUSD2 protein were selected, recloned, screened and expanded. The hybridoma clone used for these studies was 19E8. The secondary antibody was purchased from Jackson ImmunoResearch Laboratories Inc. (West Grove, PA, USA; cat. no. 112-116-143, PE conjugate).

### Formation of spheroids

Multicellular spheroids were generated using OVCAR3 and KURAMOCHI cell lines as described previously.^38^

### Mesothelial-clearance assay

Mesothelial-clearance assays were performed as described previously^9^ with minor alterations. The mesothelial cells were incubated at 37 °C for 16 h to form confluent monolayers. After the 16-h incubations, OVCAR3 and KURAMOCHI spheroids (100 cells per spheroid) were transferred to the wells containing the mesothelial monolayers.

Imaging was performed as described previously^9^ using a Nikon Ti-E Inverted Motorized Widefield Fluorescence Microscope (Nikon, Melville, NY, USA). Over 20 spheroids were imaged per condition. Phase-contrast and GFP images were captured at 0 and 24 h. The non-fluorescent surface area created by the invading spheroid in the GFP mesothelial monolayer images was measured at 24 h and divided by the initial two-dimensional area of the cancer spheroid at the initial seeding time (time 0 or 0.5 h). Twenty biological replicates were performed to calculate *P*-values for each experiment.

### IHC staining and scoring of samples

IHC analysis of HGSOC cell monolayers, OVCAR3 spheroid sections, and both TMAs were analyzed by IHC as described previously.^15^ The US Biomax array (US Biomax, Rockville, MD, USA; i.d. number: OV20814) contained 208 patient tumor samples of various ovarian malignancies, TNM and pathology grade; 16 patient samples of non-neoplastic tissues were included. A paraffin-embedded section of a benign ovary sample was obtained from the Sanford Health BioBank with institutional review board approval and patient informed consent. The other TMA was comprised of 128 cases of HGSOC patient samples; institutional review board approval and informed consent was obtained as described previously.^39, 40, 41^ The BenchMark XT automated slide staining system (Ventana Medical Systems Inc., Tucson, AZ, USA) was used for the optimization and staining of all antibodies. The Ventana iView DAB Detection Kit (Ventana Medical Systems Inc.) was used as the chromogen, and the slides were counterstained with hematoxylin. Omission of the primary antibody served as the negative control. The polyclonal anti-SUSD2 antibody was purchased from Prestige antibodies Sigma Aldrich Corp. (St Louis, MO, USA; cat. no. HPA004117). The secondary antibody was purchased from Jackson ImmunoResearch Labs (cat. no. 111065144). TMAs were chosen based on sample availability of specified subtypes of ovarian cancer.

An independent pathologist (BEH) not affiliated with the Egland Lab reviewed IHC staining of the HGSOC TMA and scored each tissue sample based on the percentage of epithelial cancer cells stained for SUSD2, on a scale of 0 to 4. The scoring distribution is defined as the following: a score of 0, no SUSD2 staining; 1, <10% positive SUSD2 staining; 2, 10–50% positive SUSD2 staining; 3, 51–75% positive SUSD2 staining; 4, >75% positive SUSD2 staining. Tissues with scores ranging from 0 to 2 were grouped together and classified as having low levels of SUSD2, whereas tissues with scores ranging from 3 to 4 were grouped together and classified as having high levels of SUSD2. An independent statistician (JFL) correlated the overall survival data from each patient to the percentage of stained SUSD2 cells in the tumor, generating a Kaplan–Meier curve.

### Cell proliferation assays

To determine cellular proliferation rates, 6 × 10^4^ OVCAR3 cells per well, 9 × 10^4^ OVSAHO cells per well and 4 × 10^5^ KURAMOCHI cells per well were plated in 12-well cell culture plates in triplicate. Cells were collected and counted every 24 h using an automated cell counter (Coulter Particle Counter, Beckman Coulter, Indianapolis, IN, USA). Three biological replicates were used for each cell line assayed.

### Migration assays

Cellular migration was analyzed using Boyden chamber-style cell culture inserts without Matrigel (BD Falcon, Corning, NY, USA) as described previously.^15^ HGSOC cells were seeded at the following concentrations: 5 × 10^5^ OVCAR3 cells per well, 1 × 10^6^ OVSAHO cells per well and 2 × 10^5^ KURAMOCHI cells per well. Culture medium with 10% fetal bovine serum was used as a chemoattractant in the lower chamber. OVCAR3 and KURAMOCHI cells were allowed to sit down and migrate for 24 h. OVSAHO cells were initially serum starved for 24 h before being plated. OVSAHO cells were allowed to sit down and migrate for 48 h. For all experiments, migrated cells were counted in 10 random fields per replicate. Nine biological replicates were used for each cell line assayed.

For wound-healing assays, silicone culture inserts (ibidi, Madison, WI, USA) were used as described previously.^42^ OVCAR3, OVSAHO and KURAMOCHI cells were seeded in each compartment of the inserts at 6 × 10^5^, 1.2 × 10^6^ and 6 × 10^5^ cells per ml, respectively. After 24 h, cell culture inserts were removed. OVCAR3, OVSAHO and KURAMOCHI cells were allowed to infiltrate the exposed wound for 20, 60 and 24 h, respectively. Images of infiltrating cells were taken every 4 h. Cell migration was quantified by measuring the area of invasion in each image captured (area was quantified using the ImageJ software, National Institutes of Health, Bethesda, MA, USA). Nine biological replicates were used for each cell line assayed.

### RT-qPCR

RT-qPCR was performed as described previously.^15^ The following primer sequences were used: TWIST1 (Twist-1)—forward, 5′-AGCTACGCCTTCTCGGTCT-3′ and reverse, 5′-CCTTCTCTGGAAACAATGACATC-3′ ZEB1 (Zeb-1)—forward, 5′-TTTTTCCTGAGGCACCTGAA-3′ and reverse, 5′-AAAATGCATCTGGTGTTCCAT-3′ CDH2 (N-cadherin)—forward, 5′-CTCCATGTGCCGGATAGC-5′ and reverse, 5′-CGATTTCACCAGAAGCCTCTA-3′; KRT14 (Keratin-14)—forward, 5′-CCTCCTCCCAGTTCTCCTCT-3′ and reverse 5′-ATGACCTTGGTGCGGATTT-3′; CDH1 (E-cadherin)—forward, 5′-TGGAGGAATTCTTGCTTTGC-3′ and reverse, 5′-CGCTCTCCTCCGAAGAAAC-3′ GAPDH—forward, 5′-AGCCACATCGCTCAGACAC-3′ and reverse, 5′-GCCCAATACGACCAAATCC-3′ SNAI3 (Snail-3)—forward, 5′-GGAGACGCAGAGAGAAATCAATG-3′ and reverse, 5′-CTTCCTCGATCCGTGGCA-3′ COL5A2 (collagen, type V, α2)—forward, 5′-GGAAGAAGACGAGGATGAAGGATA-3′ and reverse, 5′-CAGGACCAGAAGGACCAACT-3′; SNAI1 (Snail-1)—forward, 5′-GACCCACACTGGCGAGAA-3′ and reverse, 5′-GGCAGGTATGGAGAGGAAGAG-3′ AHNAK (AHNAK nucleoprotein)—forward, 5′-CTCAGGTTGTGACCGA-GATTC-3′ and reverse, 5′-CTCCTCCTTCTCCATCTTTGC-3′; and STEAP1 (six transmembrane epithelial antigen of the prostate 1)—forward, 5′-GGCATATCAACAGGTCCAACAA-3′ and reverse, 5′-ACAGCCAACAGAGCCAGTA-3′. Primers were ordered from Integrated DNA Technologies (Coralville, IA, USA). SYBR green was used with a ROX internal control reference dye. Dissociation curves were run to control for internal dimerization as well as dimerization of primers. Quantification of RT-qPCR was performed by the comparative Ct method (delta−delta Ct). Three technical replicates were performed and averaged for each cell line.

### Western immunoblot analysis

Western immunoblot analysis was performed using whole-cell lysates derived from HGSOC cell lines as previously described with minor alterations.^15^ To lyse cells grown as spheroids, 1% sodium dodecyl sulfate was added to the lysis buffer. Approximately 40 μg of protein lysate per cell line was analyzed. Equal loading was verified by incubating the membranes with anti-GAPDH antibody. Primary antibodies used include monoclonal mouse anti-GAPDH (GeneTex, Irvine, CA, USA; cat. no. GTX627408), monoclonal rabbit anti-E-cadherin (Cell Signaling, Danvers, MA, USA; cat. no. 3195), polyclonal rabbit anti-SUSD2 (Prestige Antibodies Sigma-Aldrich Corp.; cat. no. HPA004117) and polyclonal rabbit anti-N-cadherin (Cell Signaling; cat. no. 4061). Secondary antibodies used were Pierce (Thermo-Fisher Scientific, Waltham, MA, USA; cat. no. 31329) for GAPDH. Pierce cat. no. 31345 was used for N-cadherin, E-cadherin and SUSD2. Experiments were performed using three biological replicates for each cell line.

### Immunoprecipitation analysis

HGSOC cell lines grown as monolayers were subjected to immunoprecipitation using the 19E8 anti-SUSD2 antibody as described previously.^15^ Precipitated immunocomplexes were analyzed via western immunoblot as described above. Experiments were performed in triplicate for each cell line. Experiments were performed using three biological replicates for each cell line.

### Statistics

Data are expressed as mean±s.d. Where indicated, Student's *t*-test (two-tailed) was used to compare two groups. A *P*-value ⩽0.05 is considered statistically significant. Kaplan–Meier analysis was used to assess survival and the log-rank test was used to compare the survival distributions to determine statistical significance. Center values shown represent median values. Data sets subjected to pairwise comparisons performed using Student's *t*-test were checked for normality and variance among groups via calculating Pearson's coefficient of skewness (skewness coefficients fell within a range of ±0.5) as well as equality of variance analysis (*P*-values>0.5).
